# A chromophore-supported structural and functional model of dinuclear copper enzymes, for facilitating mechanism of action studies†

**DOI:** 10.1039/d1sc02593g

**Published:** 2021-08-10

**Authors:** Qiu-Cheng Chen, Natalia Fridman, Boris Tumanskii, Zeev Gross

**Affiliations:** Schulich Faculty of Chemistry, Technion-Israel Institute of Technology Haifa 32000 Israel chr10zg@technion.ac.il

## Abstract

Type III dicopper centres are the heart of the reactive sites of enzymes that catalyze the oxidation of catechols. Numerous synthetic model complexes have been prepared to uncover the fundamental chemistry involved in these processes, but progress is still lagging much behind that for heme enzymes. One reason is that the latter gain very much from the informative spectroscopic features of their porphyrin-based metal-chelating ligand. We now introduce sapphyrin-chelated dicopper complexes and show that they may be isolated in different oxidation states and coordination geometries, with distinctive colors and electronic spectra due to the heme-like ligands. The dicopper(i) complex **1-Cu2** was characterized by ^1^H and ^19^F NMR spectroscopy of the metal-chelating sapphyrin, the oxygenated dicopper(ii) complex **1-Cu2O2** by EPR, and crystallographic data was obtained for the tetracopper(ii)-bis-sapphyrin complex **[1-Cu2O2]2**. This uncovered a non-heme [Cu_4_(OH)_4_]^4−^ cluster, held together with the aid of two sapphyrin ligands, with structural features reminiscent of those of catechol oxidase. Biomimetic activity was demonstrated by the **1-Cu2O2** catalyzed aerobic oxidation of catechol to quinone; the sapphyrin ligand aided very much in gaining information about reactive intermediates and the rate-limiting step of the reaction.

## Abstract 2

Di-copper chelation by sapphyrin facilitates reaction mechanism investigations and characterization of reactive intermediates regarding biomimetic catechol oxidation.

## Graphical abstract

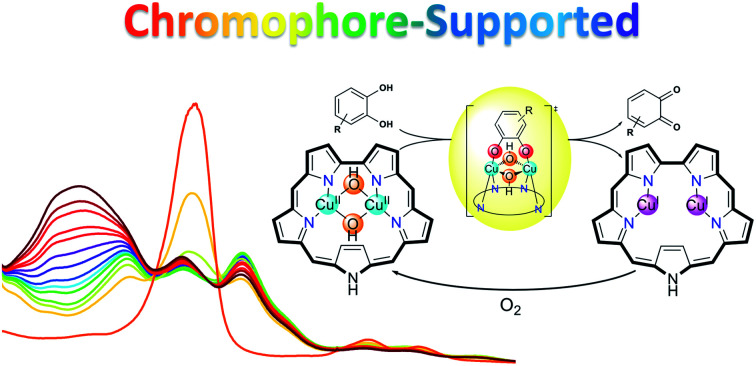

## Introduction

**Fig. 1:**
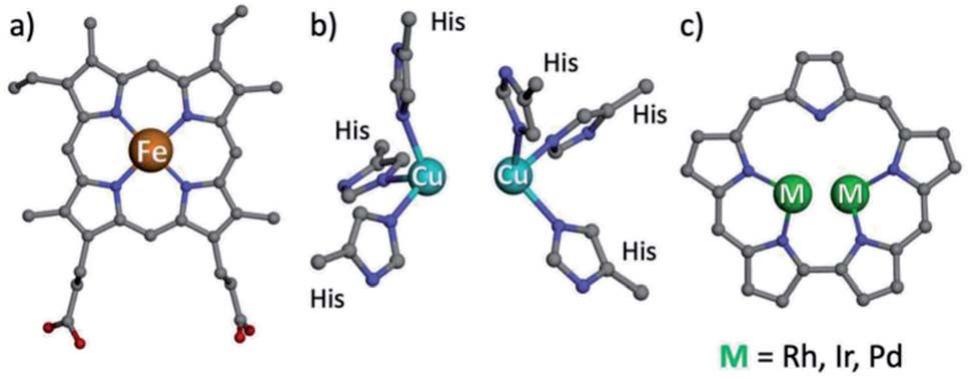
Structures of heme b (a), type III copper (b) and bimetallic sapphyrin ((c) substituents omitted).

Hemoglobin and hemocyanin share the role of dioxygen transport and have similar names, but their prosthetic groups are distinctively dissimilar: porphyrin-chelated mononuclear iron and binuclear copper, respectively (Fig. 1a and b).^1^ Both motifs are not limited to those proteins but are also present in numerous heme- and a few copper enzymes, of which catechol oxidase is the best known of the latter. It is relatively easy to study the mechanisms of heme-containing proteins/enzymes because of the strong chromophoric features of porphyrins and the facile access to synthetic derivatives required for biomimetic studies.^2,3^ Investigation of biological non-heme systems is much more demanding in general and particularly so for the type-III (T3) copper systems present in catechol oxidase.^4–9^ It is far from obvious to design biomimetics that feature the spatial distance of the two copper ions, relying on ligands whose spectroscopic features are not distinguishingly sensitive to changes during catalytic events. These include changes in the modes of both oxygen and the multidentate substrate binding to metal ions, as a function of oxidation states and many other variables.^10–15^ Even for well characterized synthetic dicopper(ii)/dioxygen complexes, the magnetic interactions between the d^9^ metal centres may differ very much regarding both the mode and the magnitude of coupling, as a function of through-space Cu–Cu distance,^16^ Cu–O–Cu bond angle,^17^ mode of dioxygen binding,^6,7,18^ protonation state of the bridging O atom(s),^19^ and many other variables.^18,20,21^ These aspects have further been shown to affect the reactivity as well, as summarized by Karlin and coworkers.^22^ Another illustrative example is the manner of catechol binding to the dicopper centre of enzymes and synthetic models catalyzing its oxidation: the most successful indications so far are based on solid state structures, while how it takes place in solution remains an open question.^5,23–27^

One possible solution for that kind of shortcoming would be using porphyrin analogs, the so-called expanded porphyrins, which are capable of binding two metal ions due to their enlarged coordination core and have sensitive absorption characteristics.^28–34^ Dicopper hexaphyrins and other porphyrinoids have been prepared for investigations that focused on spectroscopy, but all of these are neither structural nor functional models of T3 copper enzymes because the two-metal ions do not display the required cooperativity effects.^28,32,35^ Sapphyrins are the best and longest known members of expanded porphyrins (Fig. 1c), whose absorption and NMR spectra are very sensitive to structural and electronic changes.^36–39^ What is more, bimetallic sapphyrins with metal–metal distances of about 3.3 Å have been reported by Sessler, Neya and our group, but only for heavy (4d and 5d) metal ions – Rh, Ir, and Pd.^40–46^ We now report the first fully characterized 3d complexes of sapphyrins, leading to systems with highly relevant biomimetic Cu_2_O_2_ and Cu_4_O_4_ clusters, supported by a heme-like chromophore that assists the investigation of catalytic catechol oxidation reactions.

## Results and discussion

### Synthesis of Cu complexes

Screening of copper precursors and reaction conditions (Table 1) for the metalation of 5,10,15,20-pentafluorophenylsapphyrin **1-H2** (in all sapphyrin-based complexes at least one of the pyrrolic NH protons is not ionized) uncovered that: (a) no reaction occurred with the copper(ii) salts (entries 1 and 2); (b) all copper(i) salts were suitable (entries 3–8); (c) a 1 : 2 ratio of **1-H2**/[Cu] is better than an excess of Cu (entries 5 *vs.* 6); and (d) the best combination is organometallic copper and an organic base (entries 7 *vs.* 5). Under the optimized reaction conditions (entry 7), the addition of **[CuCl(BTMSA)]2** (BTMSA = bis(trimethylsilyl)acetylene) induced an immediate color change of the green **1-H2** solution to red. Column chromatography after a 30 min reaction time allowed for isolation of (the eventually characterized) **1-Cu2** in 85% yield.

**Table 1:** Initial studies and reaction optimization^a^

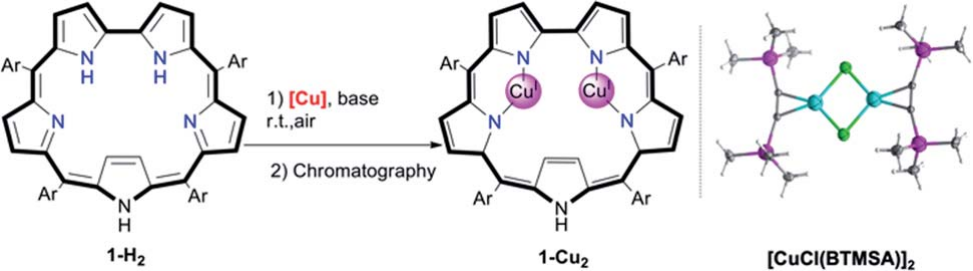				
Entry	[Cu]	Base	Solvent	Yield^b^
1	Cu(OAc)_2_	NaOAc	EtOH	—
2	Cu(acac)_2_	NaOAc	CHCl_3_	—
3	Cu(CH_3_CN)_4_PF_6_	NaOAc	CH_3_CN	12%
4	Cu(F_6_acac)BTMSA	NaOAc	CHCl_3_	65%
5	**[CuCl(BTMSA)]2**	NaOAc	CHCl_3_	76%
6^c^	**[CuCl(BTMSA)]2**	NaOAc	CHCl_3_	Trace
7	**[CuCl(BTMSA)]2**	Et_3_N	CHCl_3_	85%
8	**[CuCl(BTMSA)]2**	—	CHCl_3_	15%

a Reaction conditions: 5 mmol **1-H2**, 50 mmol base, 10 mmol [Cu], 10 mL solvent, 30 min, silica chromatography (EtOAc/*n*-hexane).

b Isolated yields.

c Excess [Cu] was used. Ar = pentafluorophenyl; BTMSA = bis(trimethylsilyl)acetylene; acac = acetylacetonato; F_6_acac = hexafluoroacetylacetonato.

**Fig. 2:**
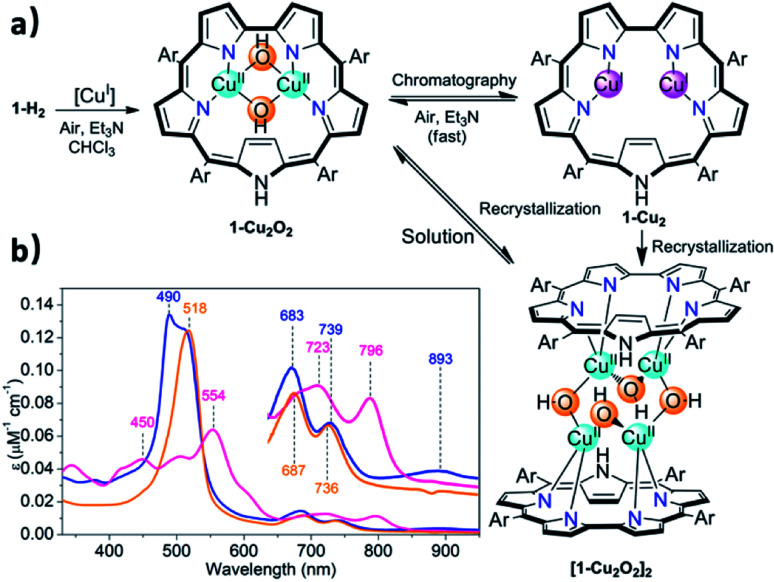
Insertion of copper into sapphyrin **1-H2** ((a) Ar = pentafluorophenyl, [Cu^I^] = **[CuCl(BTMSA)]2**), the three kinds of copper complexes obtained under various conditions, and (b) the electronic spectra of **1-Cu2O2** (orange trace), **1-Cu2** (pink trace) and **[1-Cu2O2]2** (blue trace) in CHCl_3_.

The facile insertion process allowed for *in situ* investigation of the reaction between **1-H2** and **[CuCl(BTMSA)]2** (drawing of Table 1 and Fig. S2†) in CDCl_3_, which uncovered the formation of an intermediate that is different from the final product. According to high-resolution mass spectroscopy (HRMS, Fig. S7†), the formulation is **1-Cu2O2** (Fig. 2a) and the complex has the following features: a UV-Vis spectrum with an intense Soret-like band at 518 nm and two Q-like bands at 687 and 736 nm (Fig. 2b). It has no detectable ^1^H and ^19^F NMR resonances, and its EPR spectrum (Fig. S8†) is reminiscent of that of mononuclear Cu^II^ (*S* = 1/2),^47^ surprising results that are discussed later.

Chromatographic treatment (silica: ethyl acetate/*n*-hexane) of **1-Cu2O2** yielded **1-Cu2** (*i.e.*, induced deoxygenation) according to HRMS (Fig. S6†). Consistent with that information is the ^1^H NMR spectrum that discloses the following features: (a) sharp signals consistent with the formal copper(i) oxidation state; (b) number and type of resonance coherent with the symmetry expected for the drawing presented in Table 1; (c) indication of the presence of an inverted pyrrole ring with the olefinic protons in an extremely high field (−1.56 ppm) (Fig. S4†). The **1-Cu2** to **1-Cu2O2** conversion could simply be achieved by treatment of **1-Cu2** with a base (either Et_3_N or NaOAc) and is easy to follow since its electronic spectrum is distinctively different with the maxima at 450 and 554 nm and Q-like bands that are red-shifted by 40–60 nm (Fig. 2b) relative to those of **1-Cu2O2** (detailed experimental procedures in Fig. S3†). Compared to the spectrum of free-base sapphyrin **1-H2** (Fig. S11†), both **1-Cu2O2** and **1-Cu2** have only two rather than four Q-like bands. This is reminiscent of observations for bis-palladium(ii) and bis-rhodium(i) sapphyrins and consistent with a fixed *C*_2v_ symmetry rather than a flexible symmetry due to the combination of N–H tautomerism and flipping of individual pyrrole moieties in the non-metalated sapphyrin.^42,44,45^ The apparent reason for the easy inter-conversion between the oxidized and reduced species as a function of slight variations was provided by cyclic voltammetry. There are two very close redox processes, at −0.26 and +0.11 V (Fig. S15†), suggesting that the stabilities of the reduced and oxidized complexes do not differ much.

### Structure of [**1-Cu2O2**]_2_

**Fig. 3:**
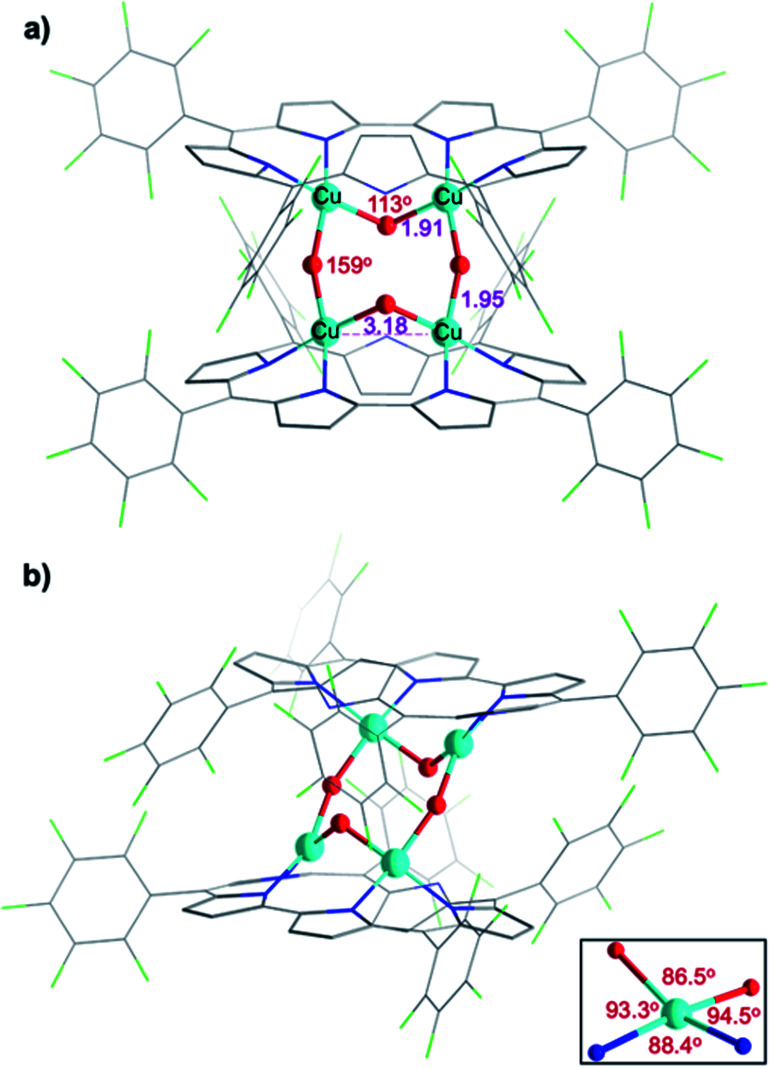
X-ray crystal structure of **[1-Cu2O2]2**. (a) Front view with emphasis on Cu–O–Cu angles and distance between two Cu atoms coordinated by the same sapphyrin. (b) Side view and coordination environment of the Cu atoms (inset).

Attempted crystallization efforts, *via* slow evaporation of aerobic solutions containing either **1-Cu2** or **1-Cu2O2**, provided the most interesting complex (Fig. 2a). The thus obtained crystals were identified by X-ray crystallography as **[1-Cu2O2]2**, wherein each copper ion is coordinated by two sapphyrin N atoms and two O atoms that bridge between the copper ions in both intra- and intermolecular fashions. The outcome is a Cu_4_O_4_ ring motif (Fig. 3) wherein each copper ion is within a slightly distorted square planar coordination sphere with a *τ*_4_ value of 0.20 (Fig. 3b, inset).^48^ The through-space distance between two Cu atoms in the same sapphyrin ligand is 3.185(2) Å, reminiscent of that variable in met-CaOx (3.0 Å) and active models.^23,49^ The Cu–N bond lengths range from 1.982(6) to 1.999(6) Å; the Cu–O–Cu angle of 113° resembles that for native T3 copper centers and reported copper–oxygen complexes.^16,17,20^ Regarding the protonation state, one criterion would be the Cu–O bond length in **[1-Cu2O2]2**: 1.907(6) and 1.948(4) Å for those bridging the copper ions bound to the same and the other sapphyrin, respectively (Fig. 3a). These values differ very much from the 1.6–1.8 Å for Cu–O in Cu–(μ-O)–Cu and the 2.1–2.7 Å in Cu–(H_2_O)–Cu moieties, but share similarity with those of Cu–(OH)–Cu motifs in *met*-bridging O atoms.^12,23,50–52^ According to XPS analysis, the copper ions are in a +2 oxidation state indeed (Fig. S10†), which together with the indications for a hydroxo bridge completes the description of **[1-Cu2O2]2** being a neutral complex with a Z-shape Cu_4_(OH)_4_ configuration.

These structural data are also helpful for understanding the earlier mentioned surprising observation of **1-Cu2O2** displaying an EPR spectrum resembling that of mononuclear copper(ii) complexes (Fig. S8†). In contrast to most dicopper(ii) centres, in both enzymes and synthetic models, **1-Cu2O2** apparently experiences neither ferro- nor antiferromagnetic interactions: it does not have a signal within the region of the half field associated with Δ*M*_s_ = 2 that would support the ferromagnetic *S* = 1 state; strong antiferromagnetic interactions may also be ruled out by the non-observation of NMR spectra. Analysis of reported hydroxo-bridged dicopper(ii) complex uncovers very large variations in the sign and values of magnetic interaction, ranging from moderately strong ferromagnetic through negligible to strongly antiferromagnetic.^16,20^ Two main variables that determine the kind and magnitude of the coupling are the through-space Cu–Cu distance and the Cu–O–Cu angle, and Koval *et al.* have introduced the ratio of these variables, Cu–O–Cu/Cu–Cu, as a criterion.^17^ Based on that and using the information from the **[1-Cu2O2]2** complex, that is, Cu–Cu = 3.18 Å and Cu–O–Cu = 113°, a value of 35 is obtained which according to the abovementioned correlation is consistent with extremely ineffective magnetic interactions. While this conclusion must still be considered tentative because it relies on bond distances and angles determined for **[1-Cu2O2]2**, which dissociates into **1-Cu2O2** in solution, it provides a good clue for the experimental results.

### T3 copper biomimetics

**Fig. 4:**
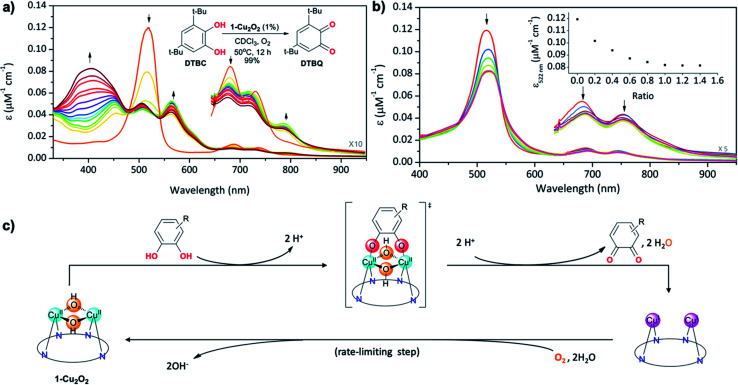
(a) Aerobic oxidation of **DTBC** to **DTBQ***via***1-Cu2O2** catalysis, monitored using changes in the absorption spectra of the latter for one hour with time intervals of 5 min (inset: reaction conditions and results obtained *via*^1^H NMR investigations). (b) UV-Vis titration of **1-Cu2O2** with increasing amounts of TCCAT and (inset) intensity changes at 518 nm as a function of the TCCAT/**1-Cu2O2** molar ratio. (c) Proposed reaction mechanism with symmetrical binding of catechol to the dicopper centre as the key intermediate and reoxidation of the reduced catalyst as the rate-limiting step.

The structural feature of the bis-copper center in the above-mentioned complexes raised the interest of exploring their potential catecholase-like catalytic activity. The most stable complex, **1-Cu2O2**, was tested as a catalyst for the aerobic oxidation of 3,5-di-*tert*-butylcatechol (**DTBC**) to 3,5-di-*tert*-butyl-*o*-benzoquinone (**DTBQ**) (Fig. 4a, inset). The conclusion from an *in situ*^1^H NMR investigation (50 °C, CDCl_3_, O_2_ purged) was that full conversion took place within 12 hours, while negligible amounts of quinone were detected without either a catalyst or O_2_. Investigations performed at much lower concentrations, *via* UV-Vis examinations, revealed that the addition of **DTBC** to **1-Cu2O2** solutions leads to very significant spectral changes (Fig. 4a). Particularly, the rise of absorption at 565 and 791 nm is clearly consistent with the formation of **1-Cu2** (pink trace in Fig. 2b). The dominance of the Cu^I^ species during catalysis suggests that its re-oxidation by O_2_ is the rate-limiting step (Fig. 4c). Regarding the fate of dioxygen, an iodide test for the possible formation of hydrogen peroxide was negative (Fig. S13†),^53^ thus pointing towards water as the product. Michaelis–Menten analysis (Fig. S12†) provided *K*_m_ = 5.6 mM and *V*_max_ = 5.2 × 10^−4^ M s^−1^, which when compared to the values obtained for catalysis by a non-aromatic macrocyclic dicopper complex (*K*_m_ = 4.9 mM; *V*_max_ = 1.3 × 10^−6^ M s^−1^)^11^ suggest similar substrate-binding strength but a much more reactive metal center in **1-Cu2O2**. Another finding was that despite the indications for **1-Cu2** being the steady state intermediate during catalysis, the β-pyrrole CH resonances of this copper(i) complex were not clearly identified by ^1^H NMR. Plausible reasons are that the bimolecular Cu(i) + O_2_ reaction is much faster at the >100 times larger concentration needed for obtaining NMR spectra, and/or also possible dynamic exchange with radical species.^54^ For distinguishing between these possibilities and also for gaining information about processes that are faster than the rate-limiting step, some focus was given to the reaction between **1-Cu2O2** and **DTBC** under anaerobic conditions and low temperature (77 K). The earlier mentioned EPR spectrum of **1-Cu2O2** changed dramatically upon the addition of **DTBC**: the broad metal signals were replaced by narrow resonances indicative of organic radicals (Fig. S9†). This is likely due to the formation of semiquinone, as reported in a more in-depth investigation that focused on the interaction of a quite similar dicopper complex with the same catechol.^54^ For testing if H-atom transfer is involved in that process, as reported for other systems,^11,25^ the rates of the **1-Cu2O2** to **1-Cu2** transformation *via* addition of **DTBC** and the deuterated compound (**DTBC-d2**) were compared. The very small deuterium kinetic isotope effect (KIE) of 1.1 (Fig. S14†) is more consistent with simple deprotonation rather than H-atom transfer from the catechol to the metal ion.

Finally, information regarding the substrate's approach to the catalyst was obtained from the titration of **1-Cu2O2** with the non-oxidizable analog tetrachlorocatechol (**TCC**). Under the same conditions that lead to the oxidation of **DTBC**, addition of **TCC** only induces intensity decrease and very small spectral shifts with no evidence for the formation of **1-Cu2** (Fig. 4b). An increase in the number of Q-like bands is expected if asymmetrical complexes are formed. However, the rather small differences relative to **1-Cu2O2** suggest that the symmetry and the coordination spheres did not significantly change in the presence of the substrate. Based on that, and also on the 1 : 1 binding affinity indicated by monitoring the absorption changes as a function of the **TCC**/**1-Cu2O2** ratio, association of the catechol with the dinuclear copper center in a symmetrical fashion is most plausible (drawing within Fig. 4c). This provides evidence for the reaction mechanism depicted in Fig. 4c: symmetrical binding of catechol to the bis-copper(ii) centre of **1-Cu2O2**, production of quinone (and H_2_O), followed by the slow aerobic Cu(i)/Cu(ii) oxidation for regeneration of the active catalyst.

## Conclusions

Sapphyrin **1-H2** is shown to be capable of chelating two copper(i) ions, forming the complex **1-Cu2**. Aerobic oxidation leads to **1-Cu2O2**, which contains a T3 copper motif supported by a heme-like ligand. Recrystallization induces the formation of the tetranuclear complex **[1-Cu2O2]2**, which was analyzed by X-ray crystallography. On top of being the first fully characterized sapphyrin complex with a first-row transition metal, it uncovers a rare Z-shaped [Cu_4_O_4_]^4+^ cluster that is clipped by two sapphyrin ligands. The Cu–(OH)–Cu motif in **[1-Cu2O2]2** is very similar to that present in *met*-catechol oxidase. Functional relevance is demonstrated as well, by showing that **1-Cu2O2** is an enzyme-like catalyst for the aerobic oxidation of **DTBC** to **DTBQ**. The advantage of the sapphyrin ligand is that all abovementioned complexes are strongly colored and have distinctively different electronic spectra. This was used for deducing that the rate-limiting step is the reaction between **1-Cu2** and dioxygen and for concluding that the catechol substrate binds to the bimetallic center in a symmetrical fashion. Taken together, the findings of this research demonstrate the potential and advantages of expanded porphyrins for constructing non-heme multi-metal models that are more convenient to study directly in solution due to the prominent spectroscopic features of the ligands.

## Data availability

Detailed experimental data is included in the ESI.† Crystallographic data for the crystal structures has been deposited at the CCDC under 2073534 for [1-Cu_2_O_2_]_2_, 2102315 for [CuCl(BTMSA)]_2_.

## Author contributions

The work was conceptualized by QC and ZG. Experiments were performed by QC, NF and BT. The first draft of the manuscript was prepared by QC and the final draft was edited by all the authors.

## Conflicts of interest

The authors declare no competing financial interest.

## Supplementary Material

SC-012-D1SC02593G-s001

SC-012-D1SC02593G-s002
