# Supplementary material for: A chromophore-supported structural and functional model of dinuclear copper enzymes, for facilitating mechanism of action studies
Source: Chem Sci. 2021 Aug 10;12(37):12445–50. doi: 10.1039/d1sc02593g (PMC8480325; doi:10.1039/d1sc02593g)
Supplement: SC-012-D1SC02593G-s001 [file SC-012-D1SC02593G-s001.pdf]

Electronic Supplementary Material (ESI) for Chemical Science.
This journal is © The Royal Society of Chemistry 2021

Supporting Information

Chromophore-Supported Structural and Functional Model of Dinuclear Copper Enzymes, for Facilitating Mechanism of Action Studies

Qiu-Cheng Chen, Natalia Fridman, Boris Tumanskii and Zeev Gross*

Schulich Faculty of Chemistry, Technion-Israel Institute of Technology, Haifa 32000, Israel.

Contents

Contents 2
Experimental Procedures 2
General methods and instruments: 2
Optimization for Cu insertion 2
Synthesis of Cu sapphyrins 3
Oxidation of **DTBC** to **DTBQ** 4
Formation of **1-Cu_2O_2** 4
NMR spectra 6
Mass spectra 7
EPR spectrum 8
XPS spectra 8
UV-vis 9
Cyclic Voltammetry 10
Crystallography 11
References 11

Experimental Procedures

General methods and instruments:

All routine chemical reagents and solvents were purchased from commercial sources and were purified by standard procedures before use. **1-H_2**[1] and **[CuCl(BTMS)]$_2$**[2] were synthesized according to literature reports. Column chromatography was performed on silica gel (Kieselgel 60, 230–400 mesh). ^{1}H and ^{19}F NMR spectra were recorded with a Bruker Avance III 400 spectrometer (400 MHz for ^{1}H and 377 MHz for ^{19}F). Chemical shifts are reported in ppm relative to the residual hydrogen atoms in $CDCl_3$ (δ = 7.24). The EPR spectra were recorded on a Bruker EMX-10/12 X-band (ν = 9.3 GHz) digital EPR spectrometer. The spectra were recorded at a microwave power of 1 mW, 100 kHz magnetic field modulation of 1 G amplitude. Digital field resolution was 2048 points per spectrum. Spectra processing and simulation were performed with the Bruker WIN-EPR. High-resolution mass spectra for the compounds were recorded with a Bruker MaXis Impact mass spectrometer. Absorption spectra were measured with an HP 8453 diode-array spectrometer. Single crystals immersed in Paratone-N oil were quickly fished with a glass rod and mounted on a Kappa CCD diffractometer under a cold stream of nitrogen at 200 K. Data collection was carried out with monochromated Mo Kα radiation using φ and ω scans to cover the Ewald sphere.[3] Accurate cell parameters were obtained with complete collections of intensities, and these were corrected in the usual way.[4] The structures were solved by SHELXS-97 direct methods[5] and refined by the SHELXL-97 program package. The atoms were refined anisotropically. Hydrogen atoms were calculated using the Riding model. X-Ray Photoelectron Spectroscopy (XPS) measurements were performed in an analysis chamber (UHV – 210^{-10} Torr during analysis) using a Versaprobe III – PHI Instrument (PHI, USA). The sample was irradiated with a Focused X-Ray AlKα monochromated X-rays source (1486.6eV) using an X-Ray beam (size 200micron, 50W, 15kV). The outcoming photoelectrons are directed to a Spherical Capacitor Analyzer (SCA). The sample charging was compensated by a Dual Beam charge neutralization based on a combination of a traditional electron flood gun and a low energy argon ion beam. Elemental analysis was performed on Thermo Scientific CHNS Analyzer (Flash2000).

Optimization for Cu insertion

A. Selectivity for metal ions

Reaction of **1-H_2** with most of the essential metal chloride salts (Na^+, K^+, Ca^{2+}, Mg^{2+}, Cr^{2+}, Fe^{2+}, Mn^{2+}, Mo^{3+}, Co^{2+}, Zn^{2+}, Ni^{2+}, Cu^{2+}, Cu^+) were carried out in DMF. 30 μL of 2 mM metal salts solutions were added into 2 mL of **1-H_2** (15 μM) solution in DMF (with excess NaOAc), whose UV-vis spectra were shown in **Figure S1**.

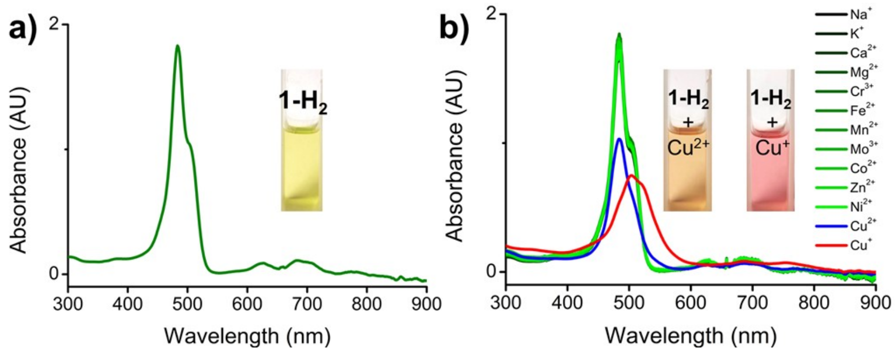

Figure S1. UV-vis spectra and pictures (insert) of (a) 15 µM **1-H$_2$** and (b) its mixture with 2 eq. metal chloride in DMF.

B. Preliminary experiments aimed at Cu insertion

The initial trials showed the high reactivity of **1-H$_2$** toward CuCl in CH_3CN or DMF in the presence of NaOAc followed by UV-vis spectroscopy, but the corresponding products could not be eluted from the column, especially after heating during the rotary evaporation. This encouraged us to continue with organic cuprous precursors for reaction in more volatile and less polar solvents. The use of $Cu(CH_3CN)_4PF_6$ in CH_3Cl with NaOAc provided a small amount of materials after chromatography, while this metal salt still suffered from slow dissolution rate. A slight improvement by replacing $CHCl_3$ by CH_3CN provide a sufficient amount of **1-Cu$_2$** for characterization. Encouraged by this preliminary result, we continued our search and finally found **[CuCl(BTMSA)]$_2$** to be the most suitable precursor for Cu insertion into **1-H$_2$**.

Synthesis of Cu sapphyrins

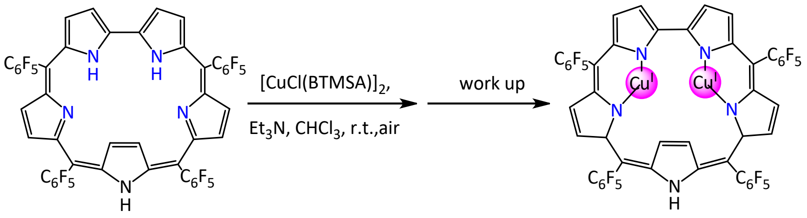

1-H$_2$ **1-Cu$_2$**

5,10,15,20-tetrakis(pentafluorophenyl)sapphyrinato biscopper(I) (1-Cu$_2$). 1-H$_2$ (5.2 mg) was dissolved in $CHCl_3$ (10 mL) with Et_3N (6.9 µL) or excess NaOAc. The green solution was stirred at room temperature until the fully dissolution of **1-H$_2$**, followed by the addition of **[CuCl(BTMSA)]$_2$** solution (5.36 mg in100 µL $CHCl_3$) in one portion. The deep red mixture was stirred for 30 minutes in an open flask and was monitored by TLC (EtOAc/n-hexane=1/9) until full consumption of the starting materials. The solvent was evaporated in vacuo without heating, and the residue was purified by chromatography on a silica column (EtOAc/n-hexane=1/9) to yield **1-Cu$_2$** as dark green solid (4.9 mg, 85%). Note: fast elution is preferred to minimize the material loss on the column. ^{1}H NMR (400 MHz, Chloroform-*d*) δ = 11.82 (s, 1H), 9.92 (d, *J*=4.5, 2H), 8.79 (d, *J*=4.3, 2H), 8.77 – 8.72 (m, 4H), -1.56 (s, 2H). ^{19}F NMR (377 MHz, Chloroform-*d*) δ = -136.92 (dd, *J*=27.5, 9.4, 2F), -137.26 (dd, *J*=26.4, 10.5, 2F), -137.87 (dd, *J*=24.1, 9.3, 2F), -138.54 (dd, *J*=27.3, 9.9, 2F), -151.28 (t, *J*=22.2, 2F), -152.84 (t, *J*=21.7, 2F), -160.01 (m, 4F), -162.07 (m, 4F).HRMS$^-$ (ACPI, negative mode) for $C_{48}H_{10}N_5F_{20}Cu_2$: m/z = 1161.9209 (calculated), 1161.9216 (observed). UV-vis ($CHCl_3$): λ_{max} (ε($M^{-1}cm^{-1}$)) = 450 (46000), 504(48000), 554 (64200), 723 (12600), 796 (11300) nm.

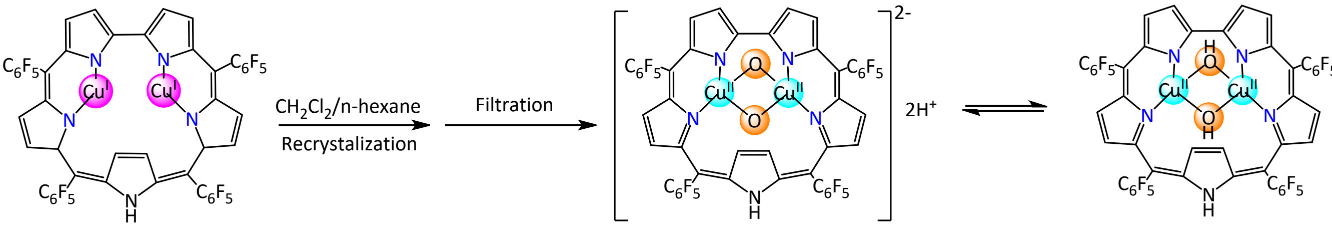

1-Cu$_2$ **1-Cu$_2$O$_2$** **1-Cu$_2$O$_2$**

5,10,15,20-tetrakis(pentafluorophenyl)sapphyrinato bis(η^2-O-copper(II)) (1-Cu$_2$O$_2$). NaOAc (5mg) or Et_3N (2 mL) was added to the solution of **1-Cu$_2$** (5.8 mg) in 10 mL $CHCl_3$ present in an open flask with stirring. The mixture was stirred for 1 hour, monitored by UV-vis and TLC (EtOAc/n-hexane=1/9) until the full consumption of the starting materials and generation of polar pink materials. The pink solution was filtered through celite, and then washed with 10 mL $CHCl_3$. The combined organic layers were evaporated at room temperature to yield **1-Cu$_2$O$_2$** in quantitative yield. HRMS$^-$ (ACPI,

negative mode) for $C_{48}H_{10}N_5F_{20}Cu_2O_2$: m/z = 1193.9107 (calculated), 1193.9080 (observed). Elemental analysis for $C_{48}H_{11}N_5F_{20}Cu_2O_2$: C 48.18, H 0.93, N 5.85 (calculated), C 48.50, H 0.97, N 5.66 (observed). UV-vis ($CHCl_3$): λ_{max} (ε($M^{-1}cm^{-1}$)) = 518 (125000), 687(12000), 736 (8400) nm.

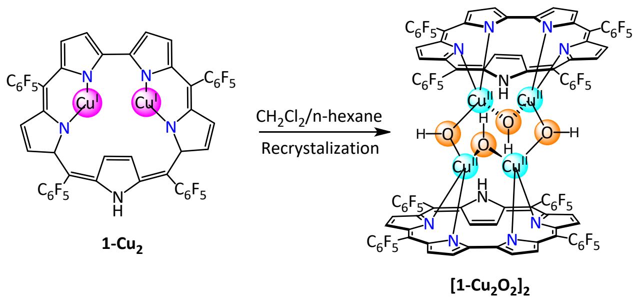

Bis(5,10,15,20-tetrakis(pentafluorophenyl)sapphyrinato)Cu_4O_4 ([1-Cu_2O_2]$_2$). Slow diffusion of layered n-hexane (1 mL) into **1-Cu_2** (2.9 mg) solution in CH_2Cl_2 (1 mL) in an open vial at 4 °C afforded dark green crystals (0.8 mg, 26%) in the bottom of the vial. ^{1}H NMR (400 MHz, Chloroform-*d*) δ = 16.70 (brs, 4H), 14.64 (brs, 4H), 13.69 (brs, 4H), 12.91 (brs, 2H), 12.29 (brs, 4H), -0.72 (brs, 4H). ^{19}F NMR (377 MHz, Chloroform-*d*) δ = -135.54 (brs, 2F), -137.73 (d, *J*=34.0, 2F), -139.54 (d, *J*=27.4, 2F), -142.29 (brs, 2F), -151.02 (t, *J*=22.8, 2F), -152.29 (t, *J*=22.0, 2F), -158.82 (brs, 2F), -161.36 (t, *J*=23.2, 2F), -161.66 – -163.20 (m, 4F). UV-vis ($CHCl_3$): λ_{max} (ε($M^{-1}cm^{-1}$)) = 490 (134000), 683(14000), 739 (8700), 893 (3700) nm.

Synthesis of [CuCl(BTMS)]$_2$

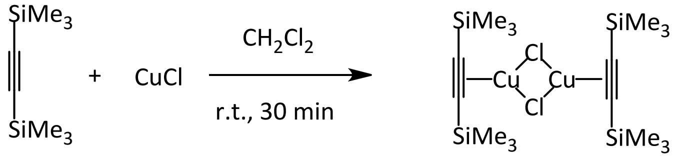

230 mg CuCl (2.35 mmol) was added to a solution of 400 mg (2.35 mmol) bis(trimethylsilyl)acetylene in 100 mL CH_2Cl_2 and stirred for 30 minutes at 25 °C. After celite filtration, the solution was concentrated to 20 mL and cooled to -17 °C. Colorless crystals were obtained after slow evaporation of the solvent under -17 °C. Characterization data according to literature.[2]

Synthesis of DTBC-d$_2$

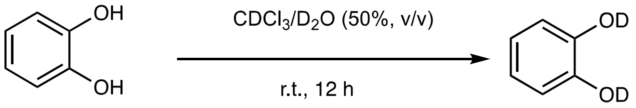

Deuterated analogue of **DTBC**, in which the protons of the phenol groups are substituted with deuterium (**DTBC-d$_2$**), was prepared by stirring the $CDCl_3/D_2O$ (50%, *v/v*) solution of 100 mg **DTBC** at rooom temperature for 12 hours. ^{1}H NMR studies indicated that 70% of the hydroxyl protons were replaced by deuterium.

Oxidation of DTBC to DTBQ

Catalytic oxidation of catechol was carried out under O_2 bubbling condition as follows: **1-Cu_2O_2** (1.193 mg, 1 μmol) was dissolved in a solution of 3 mL $CDCl_3$ containing 0.1 mmol of **DTBC** (22 mg). The ratio of the dicopper complex to catechol was 1:100. The vial was then sealed by a rubber septum, with gentle O_2 bubbling through a fine needle with another needle for outlet. The whole system was stirred and placed in 50 °C oil bath for 12 h. 1,3,5-trimethoxybenzene (16.8 mg 0.1 mmol, δ = 6.06 (s, 3H)) was then added as internal standard for yield calculation via ^{1}H NMR.

Formation of 1-Cu_2O_2

The reaction between **1-H_2** and **[CuCl(BTMSA)]$_2$** yielded an intermediate differ from the final product, **1-Cu_2**, after column purification. The ^{1}H NMR titration of **1-H_2** by **[CuCl(BTMSA)]$_2$** revealed the loss of diamagnetic resonances of the β-H on the sapphyrin ligand (**Figure S2**). This evidenced the formation of paramagnetic cupric species. Sapphyrin incorporated with two Cu and two O was detected by mass spectrometry, without any evidence of the formation of mono-copper insertion compound in any case. Together with the much higher affinity between **1-H_2** and Cu^+ instead of Cu^{2+}, this suggested the simultaneous oxidation and capture of O_2 are essential to form the stable Cu sapphyrin. The role of the base in this synthesis is elucidated by the treatment of air to the freshly prepared **1-Cu_2** (**Figure S3**). The signature maxima of **1-Cu_2** at 544 and 796 nm (**Figure 2**) disappeared completely with the raise of 518, 687 and 736 nm standing for the **1-Cu_2O_2** in the presence

of 10 eq. Et_3N and air (**Figure S3**). This transformation from the cuprous to cupric species also occurs without the involvement of base, but much slower.

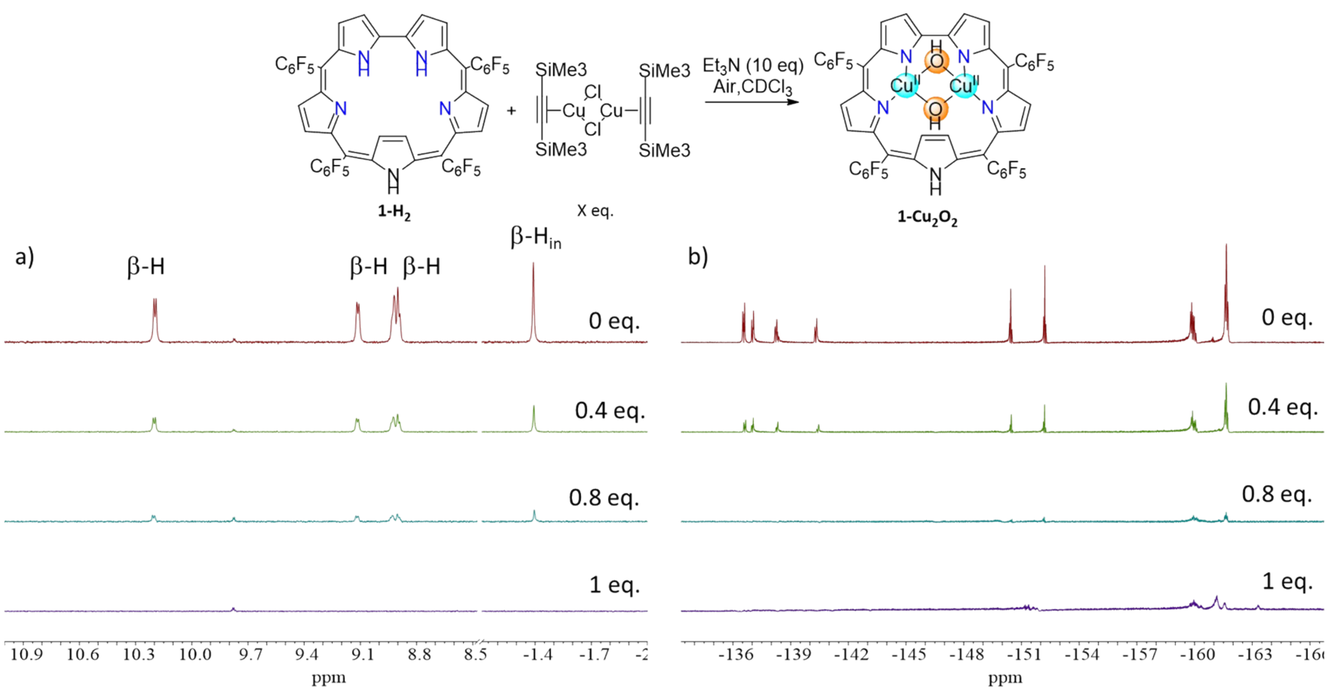

Figure S2. ^{1}H NMR (400 MHz, a) and ^{19}F NMR (377 MHz, b) titration of **1-H$_2$** by 0 to 1 eq. **[CuCl(BTMSA)]$_2$** in the presence of 10 eq. Et_3N in $CDCl_3$.

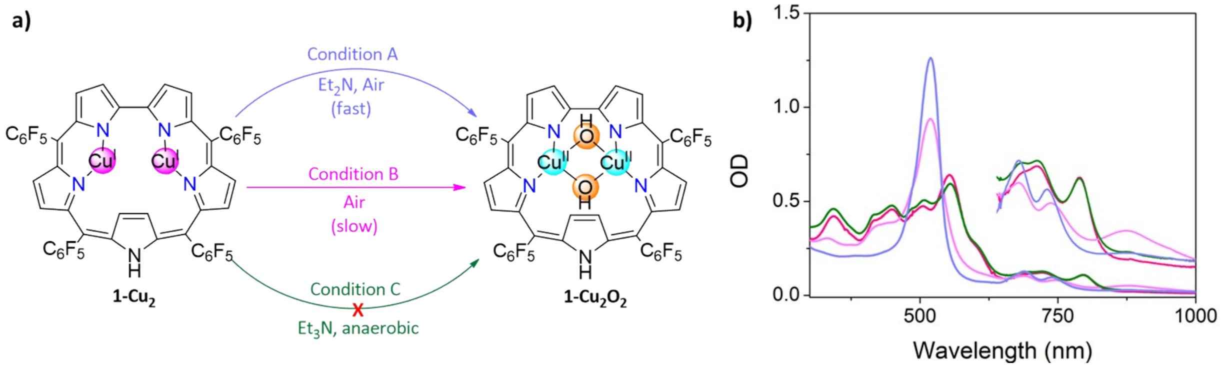

Figure S3. (a) Transformation of **1-Cu$_2$** (10 µM, $CHCl_3$, N_2, pink trace in (b)) to **1-Cu$_2$O$_2$** monitored by (b) UV-vis spectroscopy under the condition: A. 10 eq. Et_3N and air bubble (blue trace); B. air bubble (purple trace); C. 10 eq. Et_3N under N_2 (green trace).

Spectra

NMR spectra

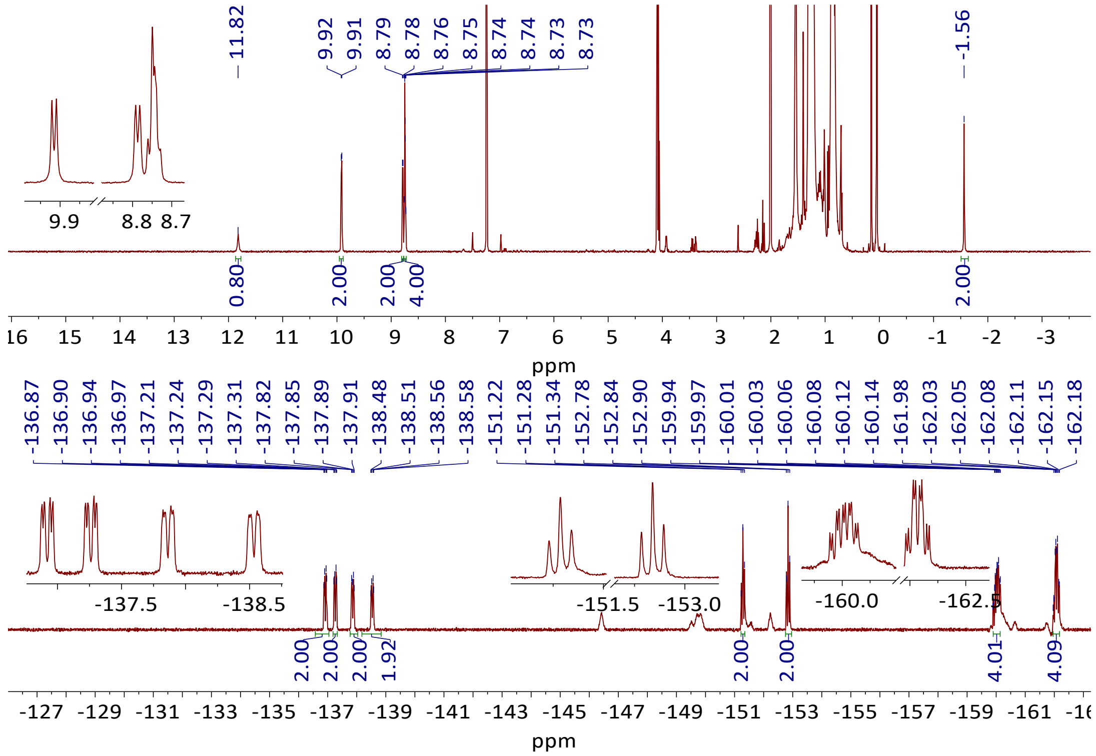

Figure S4. ^{1}H NMR (400 MHz, upper) and ^{19}F NMR (377 MHz, bottom) spectra of **1-Cu$_2$** in $CDCl_3$.

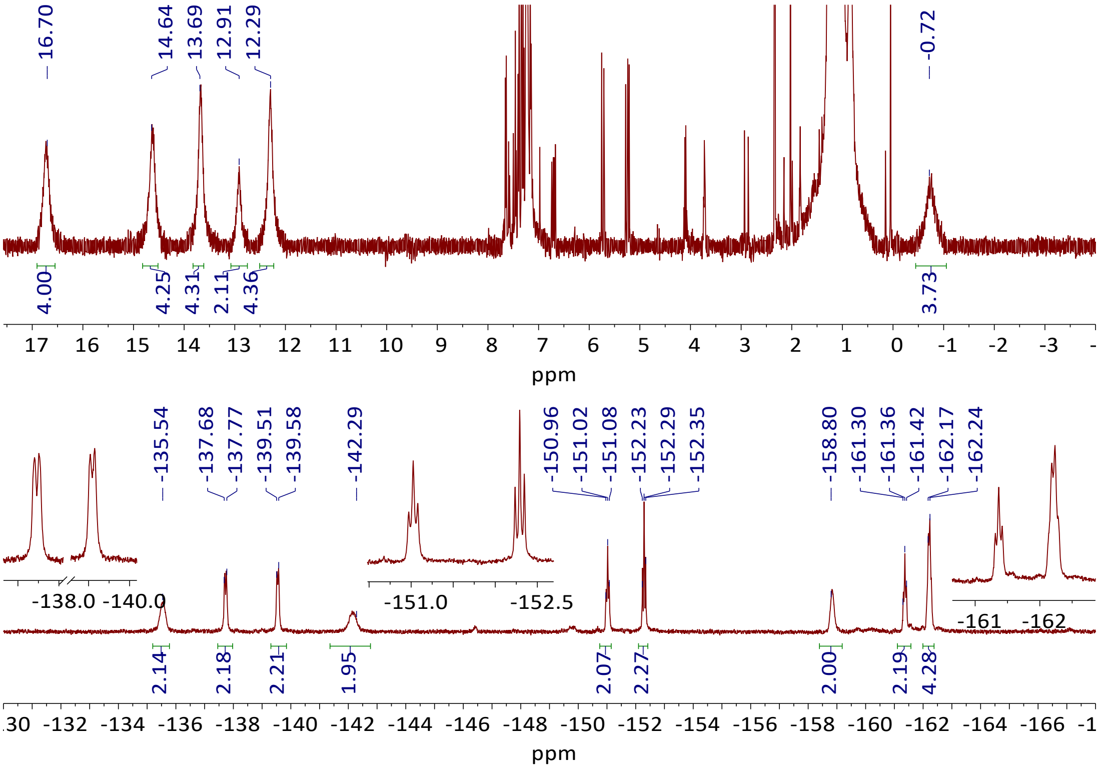

Figure S5. ^{1}H NMR (400 MHz, upper) and ^{19}F NMR (377 MHz, bottom) spectra of **[1-Cu$_2$O$_2$]$_2$** in $CDCl_3$.

Mass spectra

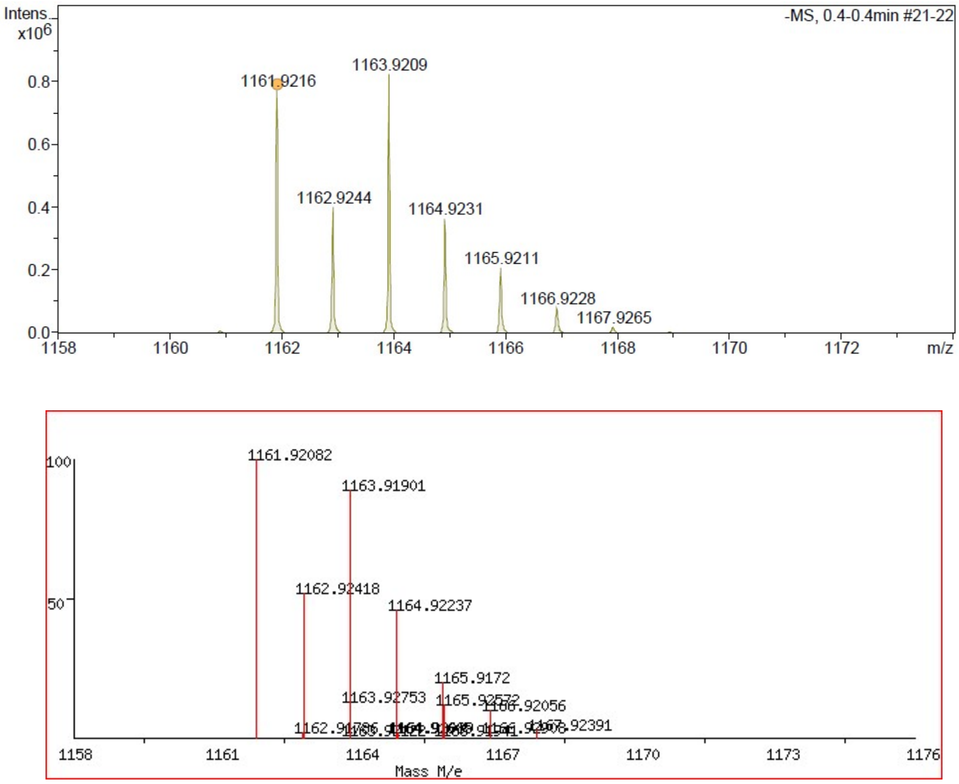

Figure S6. Experimental (top) and simulated (bottom) HRMS$^-$ (APCI) of **1-Cu$_2$**.

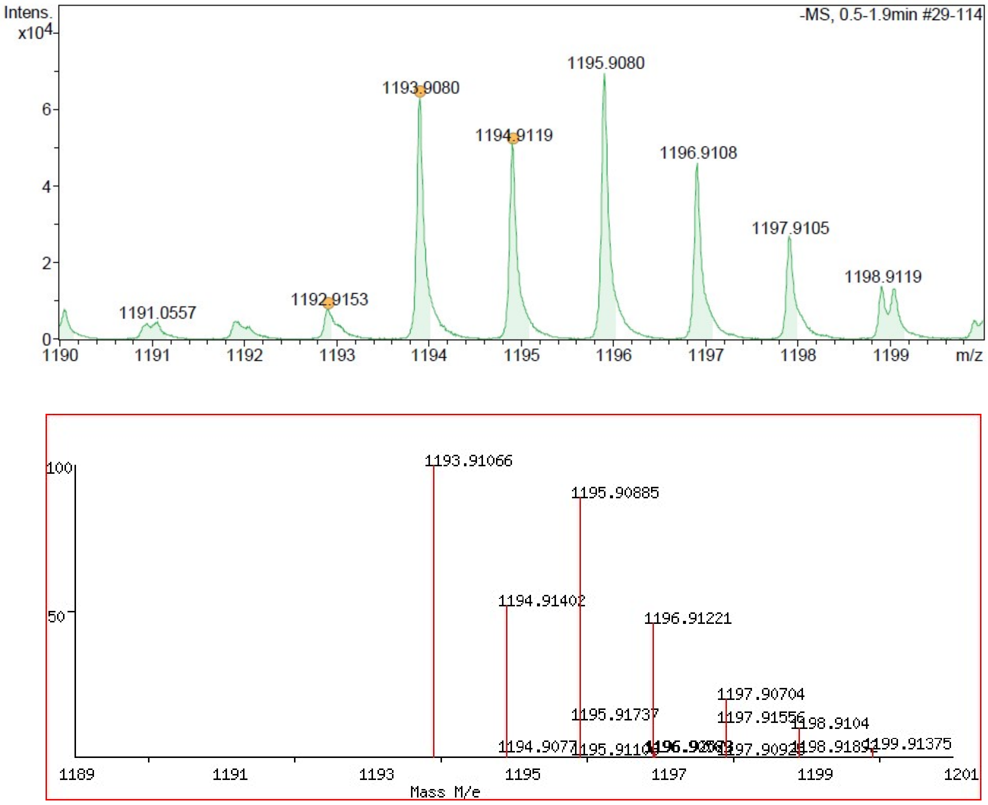

Figure S7. Experimental (top) and simulated (bottom) HRMS$^-$ (APCI) of **1-Cu$_2$O$_2$**.

EPR spectrum

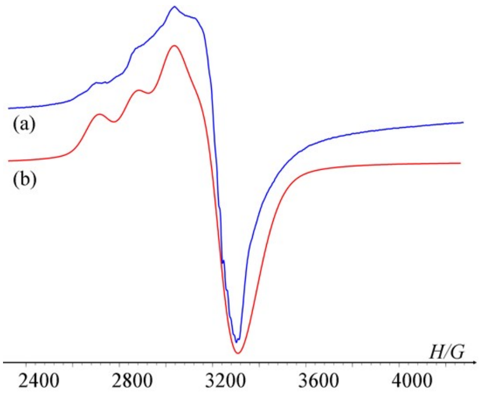

Figure S8. X-band EPR spectrum of frozen dichloroethane solution of **1-Cu_2O_2** recorded at 195 K; the red line is the simulation with parameters: $g_{\square} = 2.036$, $g_{\parallel} = 2.195$, A = 165.0G.

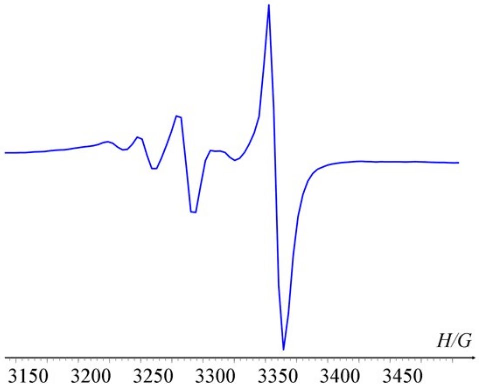

Figure S9. X-band EPR spectrum of frozen dichloroethane solution containing both **1-Cu_2O_2** and **DTBC** recorded at 77 K.

XPS spectra

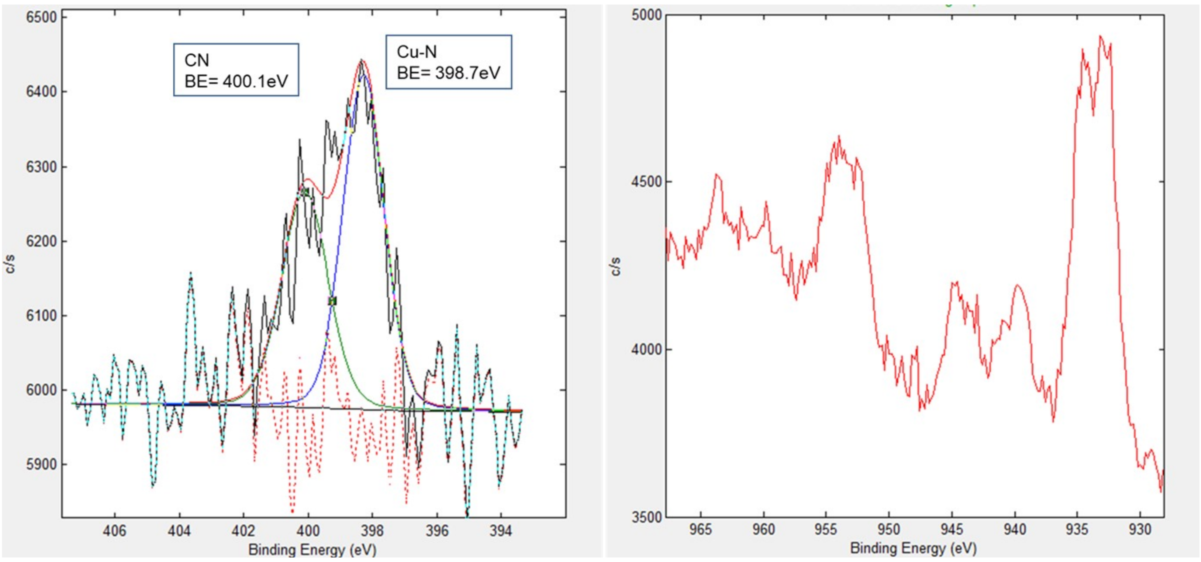

Figure S10. XPS binding energy spectra of **[1-Cu_2O_2]$_2$** on silicon wafor (a) nitrogen 1s region and (b) the copper 2p region. Peak at BE = 398.7eV, can be attributed to the Cu-N bonds. The peak at BE = 400.1eV can be attributed to C-N bonds. Strong Cu^{2+} satellites are observed, indicating the presence of the Cu^{2+} oxidation state.

UV-vis

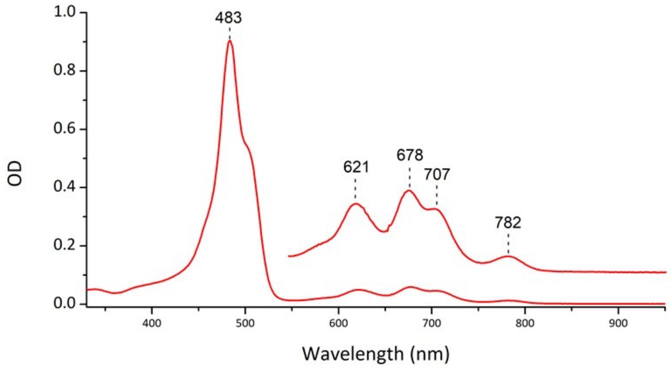

Figure S11. UV-vis spectrum of **1-H_2** (10 μM) in $CHCl_3$.

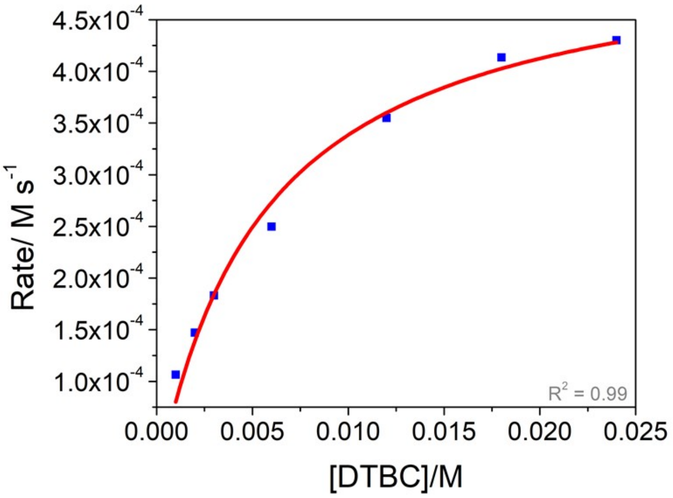

Figure S12. Plot of the substrate concentration vs. the catalytic rate for **DTBC** oxidation by **1-Cu_2O_2**, with fitting by the Michaelis–Menten equation. $V_{max} = 5.2\times10^{-4}$ M/s, $K_m = 5.6$ mM, $k_{cat} = 1.8\times10^5\ h^{-1}$.

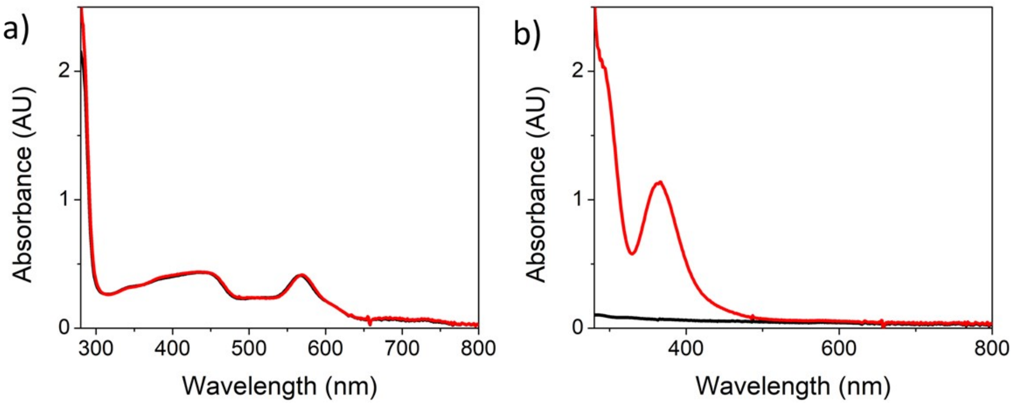

Figure S13. UV-vis spectra of (a) **DTBC** (1 mM, 10 min) oxidation catalyzed by **1-Cu_2O_2** before (black trace) and after (red trace) addition of excess TBAI (no change); and (b) UV-vis spectra of 1 mM H_2O_2 in $CHCl_3$ before (black trace) and after (red trace) addition of excess TBAI.

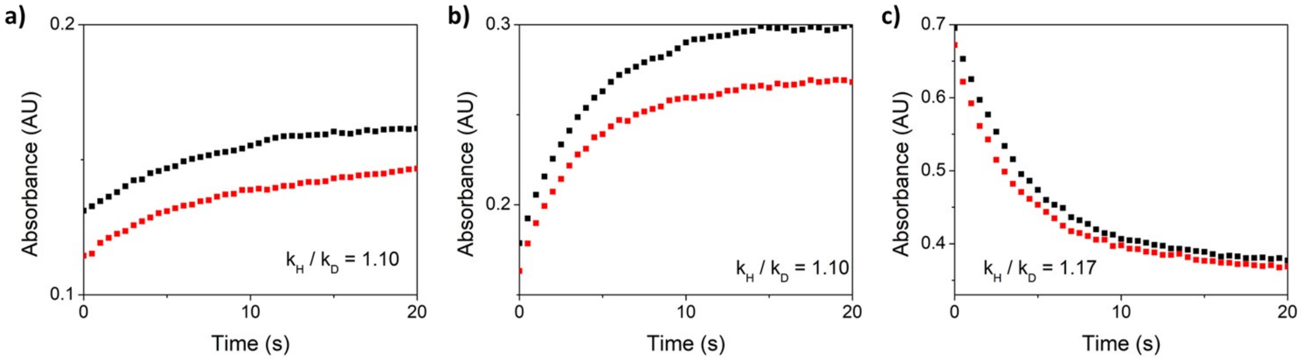

Figure S14. Plot of the absorptions vs. time for **1-Cu_2O_2** catalyzed oxidation of **DTBC** (black dots) and **DTBC-d_2** (red dots), monitored at (a) 400, (b) 565 and (c) 518 nm.

Cyclic Voltammetry

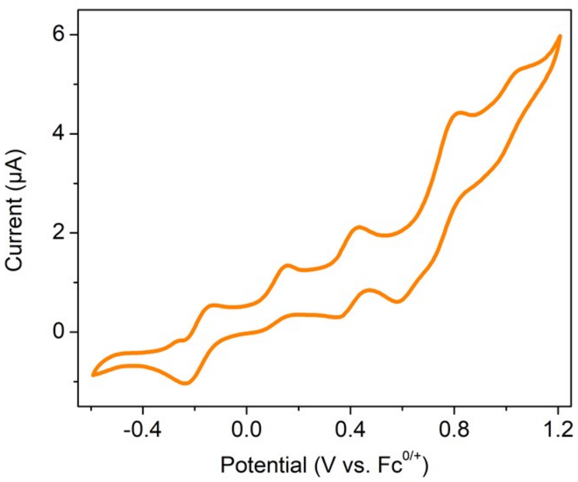

Figure S15. CV of **1-Cu_2O_2** in acetonitrile. Conditions: 0.1 M TBAP, glassy carbon working electrode, Pt wire, Ag/$AgNO_3$, r. t., N_2.

Crystallography

Table S1. Crystallographic data.

	**[1-Cu_2O_2]$_2$**	**[CuCl(BTMSA)]$_2$**
a [Å]	17.504(3)	7.8433(2)
b [Å]	21.031(3)	18.5899(5)
c [Å]	16.726(3)	21.0630(5)
a[°]	90	89.120(2)
b[°]	99.0406(4)	86.471(2)
g[°]	90	86.785(2)
V [Å^3]	6074.5	3060.25(13)
Space group	C 2/c	P -1
Z	4	2
R-Factor (%)	5.46	7.06
2θmax [°]	25.108	70.067
T (K)	200	100
CCDC number	2073534	2102315

References

(1) Simkhovich, L.; Rosenberg, S.; Gross, Z. Facile synthesis of a novel sapphyrin and its rhodium(I) complex. *Tetrahedron Lett.* **2001,** *42*, 4929-4931.
(2) Lang, H.; Herres, M.; Köhler, K.; Blau, S.; Weinmann, S.; Weinmann, M.; Rheinwald, G.; Imhof, W. Monomere alkin-stabilisierte kupfer(I)-halogenid-und kupfer(I)-pseudohalogenid-verbindungen; kristallstruktur von [(η^5-$C_5H_4SiMe_3$)$_2$Ti(C=CPh)$_2$]CuCl. *J. Organometallic. Chem.* **1995,** *505*, 85-94.
(3) Kappa CCD Server Software; Nonius BV, D., The Netherlands **1997**.
(4) Otwinowski, Z.; Minor, W. [20] Processing of X-ray diffraction data collected in oscillation mode. *Methods Enzymol.* **1997,** *276*, 307-326.
(5) Sheldrick, G. M. Phase annealing in SHELX-90: direct methods for larger structures. *Acta Cryst. A* **1990,** *46*, 467-473.
